# A mRNA panel for differentiation between acute exacerbation or pneumonia in COPD patients

**DOI:** 10.3389/fmed.2024.1234068

**Published:** 2024-03-22

**Authors:** Wilhelm Bertrams, Jochen Wilhelm, Pia-Marie Veeger, Carolina Hanko, Kristina auf dem Brinke, Björn Klabunde, Hendrik Pott, Barbara Weckler, Timm Greulich, Claus F. Vogelmeier, Bernd Schmeck

**Affiliations:** ^1^Institute for Lung Research, Philipps University Marburg, Marburg, Germany; ^2^German Center for Lung Research (DZL) Universities of Giessen and Marburg Lung Center (UGMLC), Philipps-University Marburg, Marburg, Germany; ^3^German Center for Lung Research (DZL) Universities of Giessen and Marburg Lung Center (UGMLC), Justus-Liebig-University Giessen, Giessen, Germany; ^4^Institute for Lung Health (ILH), Giessen, Germany; ^5^Department of Medicine, Pulmonary and Critical Care Medicine, University Medical Center Marburg, Philipps-University, Marburg, Germany; ^6^Center for Synthetic Microbiology (SYNMIKRO) and German Center for Infectious Disease Research (DZIF), Philipps-University Marburg, Marburg, Germany

**Keywords:** pneumonia, COPD exacerbation, comorbidity, transcriptome, COPD

## Abstract

**Introduction:**

Patients suffering from chronic obstructive pulmonary disease (COPD) are prone to acute exacerbations (AECOPD) or community acquired pneumonia (CAP), both posing severe risk of morbidity and mortality. There is no available biomarker that correctly separates AECOPD from COPD. However, because CAP and AECOPD differ in aetiology, treatment and prognosis, their discrimination would be important.

**Methods:**

This study analysed the ability of selected candidate transcripts from peripheral blood mononuclear cells (PBMCs) to differentiate between patients with AECOPD, COPD & CAP, and CAP without pre-existing COPD.

**Results:**

In a previous study, we identified differentially regulated genes between CAP and AECOPD in PBMCs. In the present new cohort, we tested the potential of selected candidate PBMC transcripts to differentiate at early time points AECOPD, CAP+COPD, and CAP without pre-existing COPD. Expression of *YWHAG*, *E2F1* and *TDRD9* held predictive power: This gene set predicted diseases markedly better (model accuracy up to 100%) than classical clinical markers like CRP, lymphocyte count and neutrophil count (model accuracy up to 82%).

**Discussion:**

In summary, in our cohort expression levels of YWHAG, *E2F1* and *TDRD9* differentiated with high accuracy between COPD patients suffering from acute exacerbation or CAP.

## Introduction

Chronic obstructive pulmonary disease (COPD) is estimated to account for ~4% of global all-cause mortality, while lower respiratory infections including community-acquired pneumonia (CAP) rank fourth (1). Exacerbations of COPD are associated with considerable morbidity and mortality (2), and increase the patient’s risk of further exacerbations (3–5). Clinically heterogeneous, the features of COPD encompass persistent airflow obstruction and predisposition to acute exacerbation (AECOPD), previously defined as an episode with acute worsening of respiratory symptoms resulting in additional therapy, most commonly triggered by respiratory tract infections (6). It is known that COPD patients have a higher risk of acquiring community acquired pneumonia (CAP) (7). CAP describes acute respiratory infection of the alveoli or the distal bronchial tree, with the term community-acquired denoting the supposed setting of pathogen acquisition. While causative treatment in form of antibiotics is readily accessible in most settings, CAP remains a high mortality disease and global health problem (6). Significant clinical interaction is seen between the entities of CAP, COPD and AECOPD. Episodes of AECOPD, which may in turn be triggered by CAP, increase the subsequent risk of future exacerbations, all the while conferring an increased risk of morbidity and mortality, driven by the coexistence of COPD and CAP (8, 9). Importantly, assessment of the risk for pneumonia in patients with COPD is crucial to determine whether inhaled corticosteroids (ICS) should be given as treatment for COPD, as ICS increase pneumonia risk in these patients (10, 11).

Both AECOPD and CAP warrant differential treatment and customization of long-term follow-up, yet clinically, AECOPD may imitate CAP in COPD patients and vice versa (12), posing an unmet need for improvement of diagnostic utilities. Furthermore, CAP might go unnoticed in COPD patients (8).

Therefore, the aim of this study was to find readily accessible candidate genes in the blood of patients that can contribute to distinguish between AECOPD, COPD+CAP and CAP only. For the establishment of candidate genes, we chose peripheral blood mononuclear cells (PBMCs). We have previously published a transcriptomic study from PBMCs in which we have identified candidate genes from PBMCs in a similar cohort based on microarray data, among them transcription factor E2F1 and YWHAG, a 14–3-3 adapter protein (13). Furthermore, we adopted DExH-box helicase/ATPase TDRD9 in the present study as potential candidate that others had identified in a study pertaining to sepsis (14).

## Methods

### Patient samples

Patients with pre-existing COPD suffering from CAP or AECOPD, and patients with CAP without pre-existing COPD were recruited on the day of or the day after hospitalization. Blood was taken immediately upon recruitment. In addition, healthy subjects were recruited as control group (Tables 1–3). Accordingly, patients were divided into four groups: (1) control group (healthy individuals), (2) CAP with pre-existing COPD (3) AECOPD (with pre-existing COPD) (4) CAP without pre-existing COPD. Group 2 and 4 were combined into one CAP group where indicated. Inclusion criteria were as published before (15) and included pulmonary infiltrates on chest x-ray and clinical presentation (CAP) and an acute respiratory worsening requiring a hospitalization in pre-diagnosed COPD but without pulmonary infiltrates on chest x-ray (AECOPD). Further CAP patients showed clinical signs or a medical history of COPD. Immunosuppressed, pregnant and HIV-positive patients were excluded from the study. The study was approved by the ethics committee of the University Medical Center Marburg (55/17). All blood donors were at least 18 years of age and provided written informed consent for use of their blood samples for scientific purposes. Peripheral blood mononuclear cells (PBMCs) were isolated by Pancoll gradient centrifugation of one collected Vacutainer EDTA-tube (6 mL whole blood). The PBMC layer was aspirated and washed 3 times at 120xg to remove platelets. Erythrocytes were lysed with red blood cell lysis buffer (Thermo Fisher Scientific). All methods were performed in accordance with the relevant guidelines and regulations as described in an earlier publication pertaining to a similar patient cohort (15).

**Table 1:** Basic characteristics of the study cohort.

	Control group (*N* = 10)	CAP (*N* = 18)	CAP+COPD (*N* = 10)	AECOPD (*N* = 18)
mean age [years ± SD]	67.1 ± 8.39	72.5 ± 16.62	69.90 ± 9.89	65.59 ± 10.16
gender m/f (%)	9/1 (90/10)	10/8 (55.56/44.44)	9/1 (90/10)	12/6 (66.6/33.3)
CRP [mg/l ± SD]	1.95 ± 0.71	107.56 ± 52.74	115.48 ± 132.79	45.21 ± 54.78
Leukocytes [WBC/nl ± SD]	7.45 ± 1.48	10.11 ± 3.86	11.08 ± 6.44	12.98 ± 3.71
BMI ± SD	ND	28.20 ± 7.71	29.64 ± 6.43	28.21 ± 5.05
Antibiotic pre-treatment [yes/no]	0/10	17/1	9/1	10/8
Steroid pre-treatment [yes/no]	1/9	1/17	4/4	12/6

**Table 2:** Severity scores of the CAP patients.

	CAP (*N* = 18)	CAP + COPD (*N* = 10)
Ø PSI score ± SD	107.94 ± 33.76	109.9 ± 36.8
PSI risk class n (%)		
I	0 (0)	0 (0)
II	2 (11.11)	2 (14.3)
III	5 (27.78)	2 (14.3)
IV	7 (38.89)	2 (14.3)
V	4 (22.22)	4 (28.76)
CURB65 score n (%)		
0	2 (11.11)	2 (14.3)
1	3 (16.67)	0 (0)
2	8 (44.44)	4 (28.6)
3	3 (16.67)	3 (21.4)
4	2 (11.11)	1 (7.1)
5	0 (0)	0 (0)
CRB-65 score n (%)		
0	1 (5.56)	2 (14.3)
1	10 (55.56)	3 (21.4)
2	3 (16.67)	4 (25.5)
3	4 (22.22)	1 (7.1)
4	0 (0)	0 (0)

**Table 3:** GOLD spirometric grades and score of the COPD patients.

AECOPD-group: GOLD classification n (%)	
I	1 (5)
II	3 (15)
III	7 (35)
IV	3 (15)
NA	4 (20)
B	4 (22)
D	13 (72)
NA	1 (6)

### RNA extraction

In order to analyze the gene expression of healthy donors, CAP, AECOPD, and CAP+COPD patients, 2*10^6 PBMCs were lysed in Trizol and RNA was purified by phenol-chloroform precipitation as described before (15). The final concentration of RNA was measured with a Nanodrop spectrophotometer.

### cDNA synthesis

RNA samples (500 ng per sample) were reverse-transcribed with random hexamer primers (HCRT Kit, ThermoFisher Scientific) for the synthesis of cDNA.

### Real-time RT-PCR

Gene expression was measured with real-time quantitative RT-PCR (StepOne, ABI Biosystems), using LUNA Universal qPCR mastermix (New England Biolabs). Each qPCR mix contained 0.1 mM forward and reverse primer and 1.5 μL cDNA in 20 μL 1x LUNA buffer. Cycling protocol was 2′ 50°C, 10′ 95°C, 40 x (15 s 95°C, 1′ 60°C). RPS18 was used as reference gene. Primer sequences are given in Table 4.

**Table 4:** Primer Sequences.

Gene	Sense (5′ → 3′)	Antisense (5′ → 3′)
RPS-18	GCGGCGGAAAATAGCCTTTG	GATCACACGTTCCACCTCATC
YWHAG	GAGCAACTGGTGCAGAAAGC	TTCGACAGTGGCTCATTCAG
TDRD9	AGTGACTGTATTGCACTTGTTGAG	CCGTCCCCAATTAAGTTCATC
E2F1	CATCCCAGGAGGTCACTTCTG	GACAACAGCGGTTCTTGCTC

Reactions were confirmed to amplify the correct products by melt curve analysis. Optimal amplification efficiencies were confirmed by the analysis of template dilution series. c_T_ values >35 were considered noise and discarded. Threshold detection was set to automatic. Δc_T_ values were calculated as c_T-Target_ – c_T-RPS18._

### Statistical analyses

Initial testing of clinical markers and candidate genes was performed in GraphPad Prism v. 6 with ANOVA and Tukey’s multiple comparison test.

Further analyses and models were done in the R programming language (v. 4.1) with packages nnet (v. 7.3–17), splines (v.4.0.1) and car (v. 3.0–12) (16–18). The predictive value of the gene expression was analyzed with multinomial models with age as co-variable. Age 70 was chosen for modeling as it approximated the mean age across all patients (Table 1). The in-sample prediction accuracy was evaluated using confusion matrices. For each test, prediction accuracy (percent of disease type correctly identified), classification accuracy (percent of correct classification for a given disease type) and overall model accuracy are indicated.

## Results

In the present study, we characterized the patients listed in Tables 1–3 and achieved a certain degree of distinction between the disease phenotypes on the basis of CRP, lymphocyte count and neutrophil count. In order to further support the diagnostic potential of these markers, we aimed to find candidate genes that help to predict a certain disease phenotype. Among the 14 tested mRNAs in our panel that were screened by qPCR in all PBMC samples (Table 5), we found three candidates with global discriminatory potential as assessed by ANOVA, while all other candidates did not show significant differences between the disease groups. YWHAG and E2F1 have been previously described by us to distinguish CAP from AECOPD (15), and TDRD9 has been shown to be of diagnostic value in a setting of sepsis (14). Therefore, the current study validates and expands the candidate genes that help to diagnose lung disease.

**Table 5:** Potential mRNA biomarkers analyzed for this study.

Analyst
YWHAG
TDRD9
E2F1
IFIT5
DYRK2
AHNAK
ARL14EP
MDC1
ADGRE3
BPGM
circ00206579
GADD45A
TAP2
CCNB1IP1

**Figure 1:**
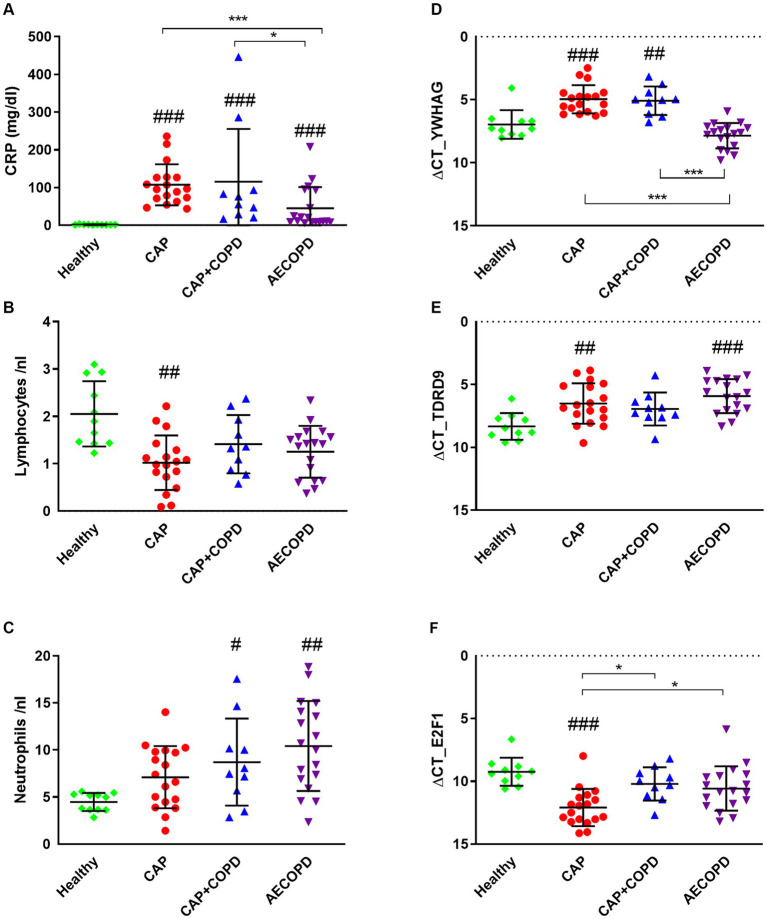
Clinical parameters and candidate gene expression were determined for all patients. Patient blood samples were tested for CRP, lymphocyte count and neutrophil count **(A–C)**. PBMC RNA samples were tested for the potential biomarkers YWHAG, TDRD9 and E2F1. Expression was determined by qPCR and differential gene expression is displayed as ΔcT value. The Y axis is inverted for a more intuitive data representation **(D–F)**. Significance was assessed on log2 transformed data by one-way ANOVA with Tukey’s correction. *p < 0.05; **p < 0.01; ***p < 0.001 (compared to indicated cohorts, # compared to healthy controls. nHealthy = 10, nCAP = 18, nCAPCOPD = 10, nAECOPD = 18).

We recruited patients with pre-existing COPD into those exclusively suffering from acute exacerbation, and those with concomitant infection of the alveolar compartment (CAP+COPD), and juxtaposed them to patients suffering only from parenchymal infection with no pre-existing condition (CAP) or healthy donors. At the end of hospital treatment we re-evaluated the patients´ classification. Classical clinical parameters CRP, lymphocyte count and neutrophil count were of limited use to differentiate diseases (Figures 1A–C). In contrast, expression of YWHAG was higher in CAP and CAP+COPD patients compared to AECOPD patients (*p* < 0.001) as well as to healthy donors (*p* < 0.01). TDRD9 was lower in healthy donors vs. AECOPD and CAP patients (*p* < 0.01). E2F1 was higher in healthy donors vs. CAP patients (*p* < 0.001) and also differentiated CAP vs. CAP+COPD patients (*p* < 0.05; Figures 1D–F).

**Figure 2:**
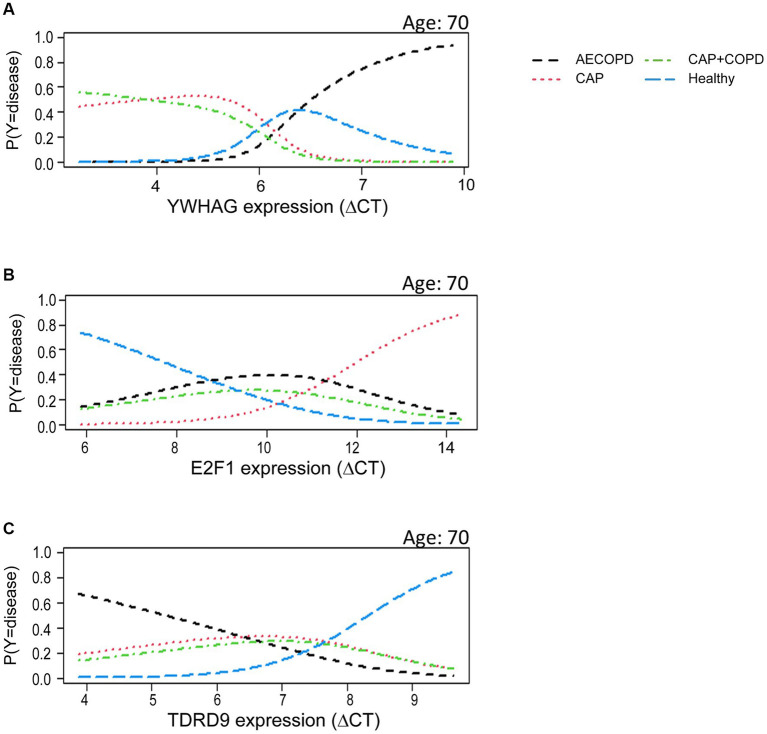
Candidate gene expression helped to predict disease. The linear model dependent on YWHAG **(A)**, E2F1 **(B)** or TDRD9 **(C)** assigns a likelihood for a given disease as a function of gene expression. The contribution of the expression information of each single gene in the multinominal model was statistically significant (*p* < 0.001, likelihood ratio test). Y is the mulinominal random variable coding the disease state. P (Y = disease) indicates the expected probability of a patient having the disease, conditional on age and target gene expression.

We constructed multinomial models to test to what extent the different disease types can be explained by expression of the selected candidate genes. This approach combines the discriminatory potential of each gene with the others, allowing for mutual complementation. Exemplarily, using YWHAG as candidate gene, in a group of modeled age = 70, high Δc_T_ values of YWHAG were associated with a high predicted probability of the subject having AECOPD (Figure 2A). High Δc_T_ values for E2F1 were associated with a high predicted probability of the subject suffering from CAP (Figure 2B). High Δc_T_ values of TDRD9 resulted in a high predicted probability of the subject being healthy (Figure 2C).

**Figure 3:**
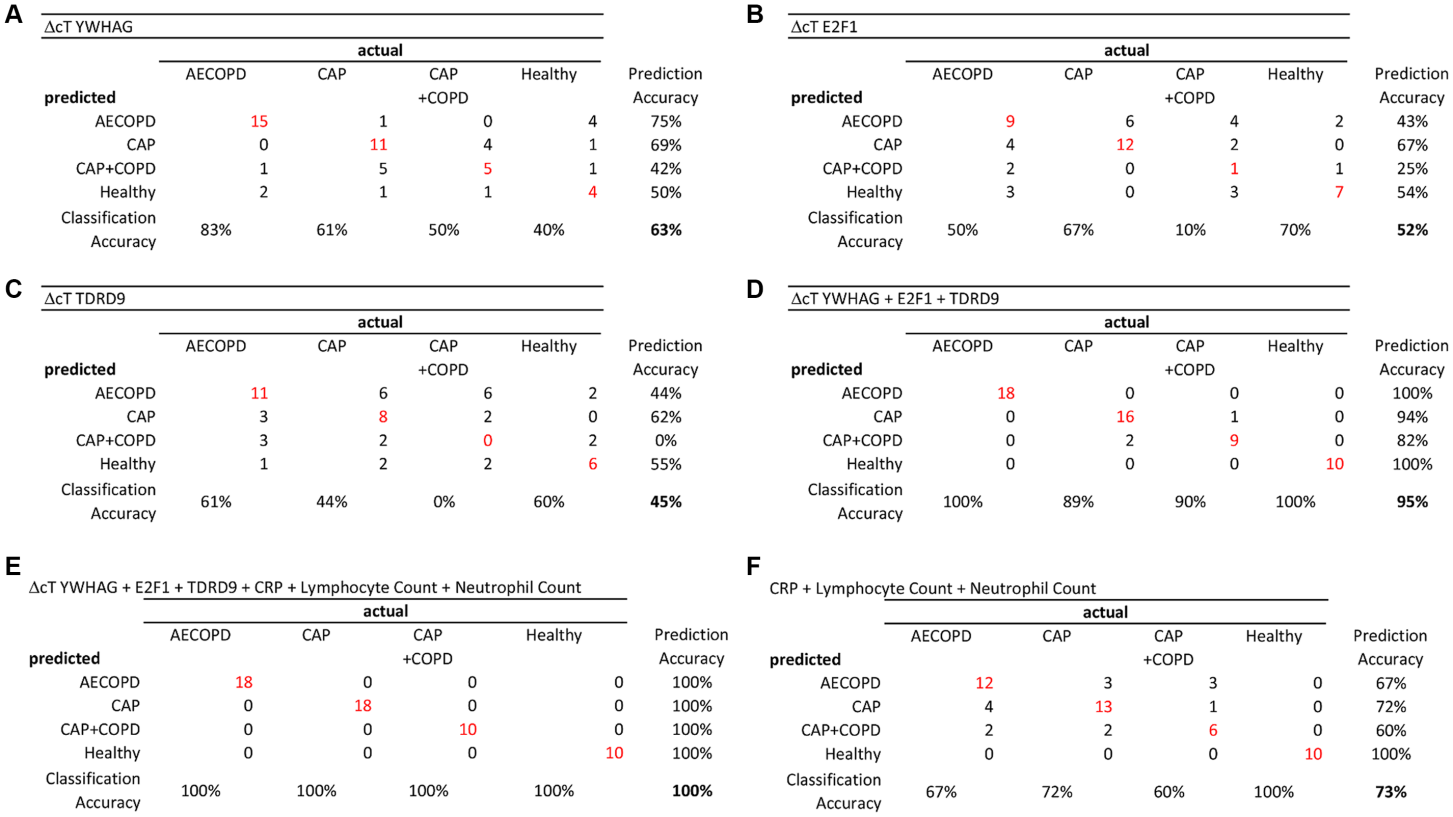
Candidate gene expression helped to diagnose a disease. The in-sample prediction accuracy was evaluated using confusion matrices. While each candidate gene alone was a not a good predictor of disease state (Model Accuracy **A**: 63%, **B**: 52%; **C**: 45%), their combination achieved markedly better prediction (**D**: 95%), considerably better than the combined clinical markers CRP, Lymphocyte count and neutrophil count (**F**: 73%). The best prediction was achieved when candidate genes and clinical markers were combined (**E**: 100%).

After establishing that only expression of the tested factors *YWHAG*, *E2F1* and *TDRD9* was generally suitable for disease prediction, we performed a systematic comparison of these candidate genes and clinical markers with confusion matrices to test their ability to help diagnose a disease (Figures 3A–C). This revealed that accurate predictions were made by the model when all three candidate genes were used as combined predictors (model accuracy: 95%, printed in bold; Figure 3D; Table 6). This could be further enhanced by combining all three candidate genes with the clinical markers, which lead to a model accuracy of 100% (Figure 3E; Table 6). Clinical markers alone did not yield an overall good prediction of disease state, with a model accuracy of 50–77% (Figure 3F; Supplementary Figure S2).

**Table 6:** Model performances on the basis of 4 (AECOPD, CAP, CAP+COPD, Healthy) or 3 (AECOPD, CAP_Total_, Healthy) health states.

Predictor	Performance	
	4 disease groups	3 disease groups
Δc_T_ YWHAG	63%	79%
Δc_T_ E2F1	52%	79%
Δc_T_ TDRD9	45%	63%
Δc_T_ YWHAG + Δc_T_ E2F1 + Δc_T_ TDRD9	95%	100%
CRP + Lymphocyte Count + Neutrophil Count	73%	82%
Δc_T_ YWHAG + Δc_T_ E2F1 + Δc_T_ TDRD9 + CRP + Lymphocyte Count + Neutrophil Count	100%	100%
CRP	71%	84%
Lymphocyte Count	50%	64%
Neutrophil Count	55%	63%

Detailed information is provided in Figure 3; Supplementary Figures S3–S5.

When CAP and CAP+COPD patients were combined into one group, clinical marker comparison yielded good separation of disease groups versus healthy donors (CAP_Total_; Supplementary Figure S1), but did not perform well in discriminating among disease groups. In the predictive model, 100% accuracy was achieved when all three candidate genes were used. Accuracy remained at this level upon inclusion of the classical clinical markers (Supplementary Figures S3, S4).

## Discussion

The aim of this study was the identification of easily accessible candidate transcripts in the blood of COPD patients suffering from an AECOPD or CAP, as accurate and early distinction of AECOPD and CAP is urgently needed to inform treatment decisions and customization of long-term follow-up. The distinction between CAP and AECOPD based on symptoms and X-ray imaging can be challenging, not least owing to the potential similarity of the associated radiological morphi (dirty chest (19) vs. infiltrates) but also because it is an unresolved question whether AECOPD is a cause or early state of pneumonia in COPD patients or an entirely separate disease entity (20). While C-reactive protein (CRP) (21) and procalcitonin (PCT) are established markers to demarcate CAP, concerns about specificity and prognostic value have been voiced (22). Notably, the array of molecular tools at hand to diagnose a disease is rapidly expanding. RNA as used in our study has also been applied to predict lung cancer (23). Recently, advanced proteomics enabled the use of new metabolic biomarkers (24) that have also been applied to diagnose COPD exacerbations (25).

Using our own (15) and other studies (14, 26) as a resource, we identified 14 potential candidate genes, and we could show, by qPCR, that three of them (YWHAG, E2F1, and TDRD9) differentiated the disease phenotypes CAP, AECOPD, CAP+COPD and healthy. E2F1 is a transcription factor with proliferative capacities, and it is found in the vascular remodeling that is associated with COPD (27). YWHAG (tyrosine 3-monooxygenase/tryptophan 5-monooxygenase activation protein gamma, 14–3-3γ) is a factor that has been shown to be a microRNA target in COPD by us and others (28), as well as in non-small cell lung cancer (29). It belongs to the 14–3-3 protein family, which can regulate signal transduction by binding to phosphoserine-containing proteins (13). TDRD9 (Tudor domain containing protein 9) is a RNA helicase that is typically germ-line associated, but has been shown to be a unfavorable prognostic marker in lung adenocarcinoma (30). While the precise functional contribution of these markers in their respective disease entities remains elusive, we highlight their diagnostic potential.

We furthermore complemented the classical clinical markers CRP, neutrophil count, and lymphocyte count with the expression data of the three RNA biomarkers, and we could show that prediction of the disease benefits from addition of E2F1, YWHAG and TDRD9, as prediction performance of the established clinical markers alone was poor.

A limitation of our study is the gender imbalance in the control and CAP+COPD group. As these groups contained with one exception only male subjects, we cannot rule out that some of our results arise from a gender-specific reaction to the disease. For this reason, we do not stratify our patients into gender groups.

This study is intended to validate the diagnostic value of the expression of these genes that have been selected based on data from a previous exploratory study (15). We were able to use a convenience sample in our hands to show that the hypothesized effects are replicable. This small sample size is independent of the samples used in the previous study. It may not be sufficient to ultimately claim the diagnostic value, but it adds relevant information and strengthens the prospect of a potential usefulness.

We propose to test for YWHAG, E2F1 and TDRD9 early-on in order to tailor the subsequent treatment to the specific patient’s needs. Our study provides a parameter set with very high prediction accuracy, and corroborates our earlier findings (15). To test whether these findings are applicable outside of our past and present patient group, our data needs to be validated with a larger cohort.

## Data availability statement

The raw data supporting the conclusions of this article will be made available by the authors, without undue reservation.

## Ethics statement

The studies involving humans were approved by Ethics committee of the University Medical Center Marburg. The studies were conducted in accordance with the local legislation and institutional requirements. The participants provided their written informed consent to participate in this study.

## Author contributions

WB and JW performed the analyses. P-MV, CH, KB, and BK performed the experiments. HP, BW, TG, and CV provided patient material and clinical data. BS supervised the study. All authors proofread the manuscript, contributed to the article, and approved the submitted version.
