# Supplementary material for: A mRNA panel for differentiation between acute exacerbation or pneumonia in COPD patients
Source: Front Med (Lausanne). 2024 Mar 22;11:1234068. doi: 10.3389/fmed.2024.1234068 (PMC10995291; doi:10.3389/fmed.2024.1234068)
Supplement: Supplementary file 1 [file Data_Sheet_1.docx]

Supplemental Methods

## Prediction of disease as a function of age, sex, and candidate gene expression

R model formulas used to fit the multinomial models for disease prediction:

Models with adjustment for age and sex:

(1) disease ~ ns(age,2) + YWHAG

(2) disease ~ ns(age,2) + E2F1

(3) disease ~ ns(age,2) + TDRD9

(4) disease ~ ns(age,2) + YWHAG + E2F1 + TDR9

Models with adjustment for age and sex including clinical markers:

(5) disease ~ ns(age,2) + CRP + Lymphocyte_count + Neutrophil_count

(6) disease ~ ns(age,2) + CRP + Lymphocyte_count + Neutrophil_count + YWHAG + E2F1 + TDR9

Models without adjustment for age and sex:

(7) disease ~ YWHAG + E2F1 + TDR9

(8) disease ~ CRP + Lymphocyte_count + Neutrophil_count + YWHAG + E2F1 + TDR9

where "disease" is a factor with levels AECOPD, CAPCOPD, CAP and Healthy, "age" is a numeric vector with patient's age in years (fitted on a natural spline basis with 2 degrees of freedom), "sex" is a factor with levels male and female, and YWHAG, E2F1, TDR9 are numeric vectors with the Δc_T_ values of the respective target genes.

## R Session Info

R version 4.0.1 (2020-06-06)

Platform: x86_64-w64-mingw32/x64 (64-bit)

Running under: Windows 10 x64 (build 19044)

Matrix products: default

Random number generation:

RNG: Mersenne-Twister

Normal: Inversion

Sample: Rounding

locale:

[1] LC_COLLATE=German_Germany.1252 LC_CTYPE=German_Germany.1252 LC_MONETARY=German_Germany.1252

[4] LC_NUMERIC=C LC_TIME=German_Germany.1252

attached base packages:

[1] splines grid stats graphics grDevices utils datasets methods base

other attached packages:

[1] tidyr_1.2.0 gtsummary_1.5.2 nnet_7.3-17 tigerstats_0.3.2 abd_0.2-8

[6] mosaic_1.8.3 ggridges_0.5.3 mosaicData_0.20.2 ggformula_0.10.1 ggstance_0.3.5

[11] dplyr_1.0.8 Matrix_1.2-18 ggplot2_3.3.5 lattice_0.20-41 nlme_3.1-148

[16] jsonlite_1.7.3 callr_3.7.0 webshot_0.5.3

loaded via a namespace (and not attached):

[1] fs_1.5.2 usethis_2.1.5 devtools_2.4.3 rprojroot_2.0.2 tools_4.0.1

[6] backports_1.4.1 utf8_1.2.2 R6_2.5.1 DBI_1.1.2 colorspace_2.0-2

[11] withr_2.4.3 gridExtra_2.3 tidyselect_1.1.1 prettyunits_1.1.1 processx_3.5.2

[16] leaflet_2.1.1 compiler_4.0.1 cli_3.2.0 gt_0.6.0 htmlTable_2.4.0

[21] desc_1.4.0 ggdendro_0.1.23 mosaicCore_0.9.0 scales_1.1.1 checkmate_2.0.0

[26] readr_2.1.2 stringr_1.4.0 digest_0.6.29 pkgconfig_2.0.3 htmltools_0.5.2

[31] manipulate_1.0.1 sessioninfo_1.2.2 labelled_2.9.0 fastmap_1.1.0 htmlwidgets_1.5.4

[36] rlang_1.0.2 rstudioapi_0.13 farver_2.1.0 generics_0.1.2 crosstalk_1.2.0

[41] zip_2.2.0 magrittr_2.0.3 Rcpp_1.0.7 munsell_0.5.0 fansi_0.5.0

[46] lifecycle_1.0.1 stringi_1.7.3 MASS_7.3-51.6 brio_1.1.3 pkgbuild_1.3.1

[51] plyr_1.8.6 ggrepel_0.9.1 forcats_0.5.1 crayon_1.4.2 haven_2.4.3

[56] hms_1.1.1 knitr_1.39 ps_1.6.0 pillar_1.7.0 pkgload_1.2.4

[61] glue_1.6.2 broom.helpers_1.6.0 remotes_2.4.2 BiocManager_1.30.16 tzdb_0.2.0

[66] png_0.1-7 vctrs_0.4.1 tweenr_1.0.2 testthat_3.1.2 gtable_0.3.0

[71] purrr_0.3.4 polyclip_1.10-0 assertthat_0.2.1 cachem_1.0.6 xfun_0.29

[76] ggforce_0.3.3 openxlsx_4.2.5 broom_0.7.12 tibble_3.1.6 memoise_2.0.1

[81] ellipsis_0.3.2

Supplemental Figure Legends:

Figure S1: Clinical parameters and candidate gene expression were determined as in Figure 1 with a combined CAPTotal group. Patient blood samples were tested for CRP, lymphocyte count and neutrophil count (A-C) and PBMC RNA samples were tested for the potential biomarkers YWHAG, TDRD9 and E2F1 (D-F) as shown in Figure 1. CAP and CAPCOPD patients were clustered into a single CAPTotal group. Significance was assessed on log2 transformed data by one-way ANOVA with Tukey’s correction. *, P < 0.05; **, P < 0.01; ***, P < 0.001; (compared to indicated cohorts, # compared to healthy controls. nHealthy=10, nCAPTotal=28, nAECOPD=18).

Figure S2: Prediction performance of classical clinical markers was moderate. The in-sample prediction accuracy was evaluated as in Figure 3 and ranged from 50 – 77 %.

Figure S3: Candidate gene expression helped to diagnose AECOPD vs. CAPTotal vs. Healthy. While each candidate gene alone was a not a good predictor of disease state (Model Accuracy A: 79%, B: 79%; C: 63%), their combination achieved markedly better prediction (D: 100%), considerably better than the combined clinical markers CRP, Lymphocyte count and neutrophil count (E: 82%). Combined candidate genes and clinical markers performed equally to the combination of candidate genes alone (F: 100%). The in-sample prediction accuracy was evaluated as in Figure 3.

Figure S4: Prediction performance of classical clinical markers to diagnose AECOPD vs. CAPTotal vs. Healthy was moderate. The in-sample prediction accuracy was evaluated as in Figure 3 and ranged from 63 – 86%.
