# Supplementary figures and images for: A mRNA panel for differentiation between acute exacerbation or pneumonia in COPD patients
Source: Front Med (Lausanne). 2024 Mar 22;11:1234068. doi: 10.3389/fmed.2024.1234068 (PMC10995291; doi:10.3389/fmed.2024.1234068)

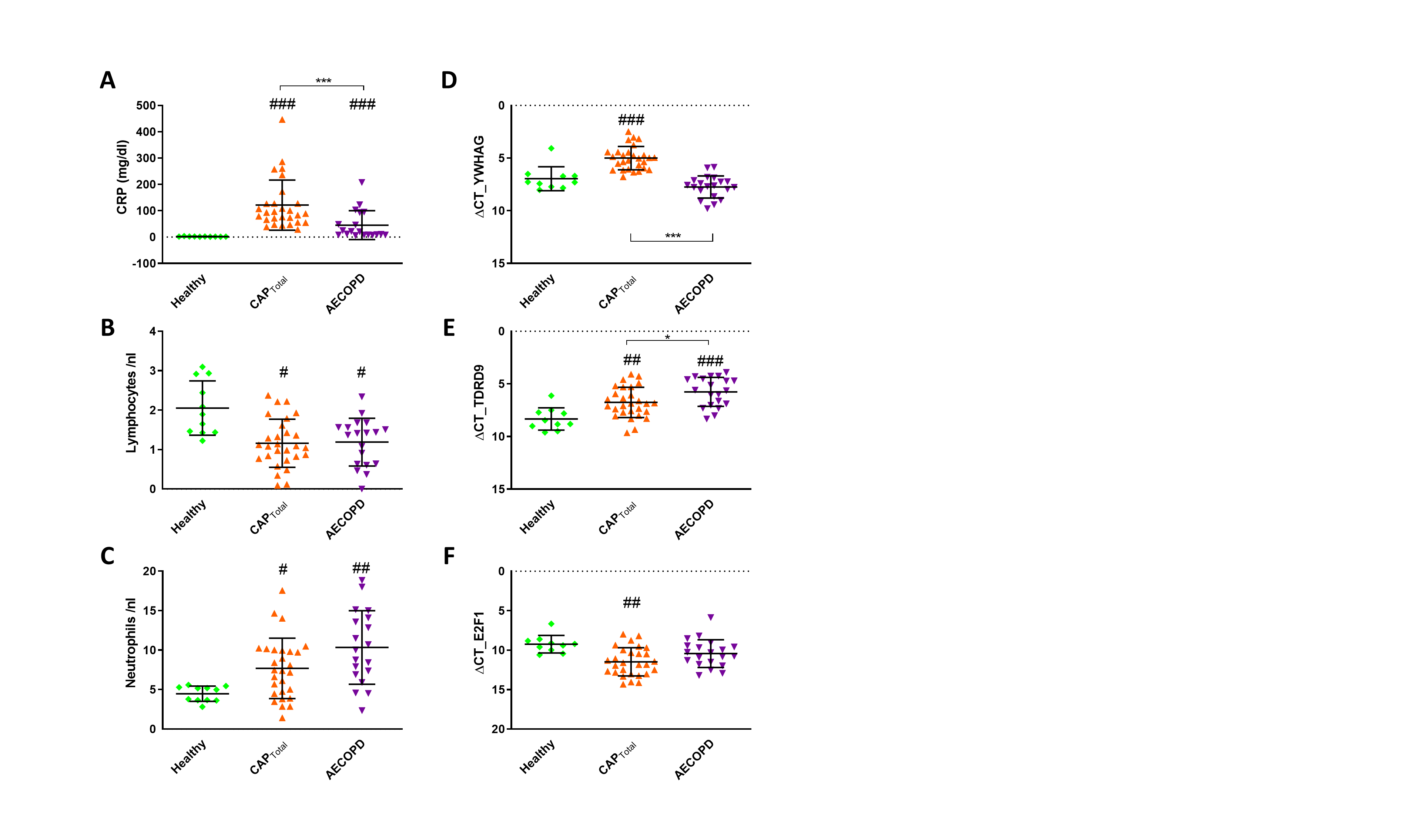

Supplement: Supplementary file 2 [file Image_1.TIF]

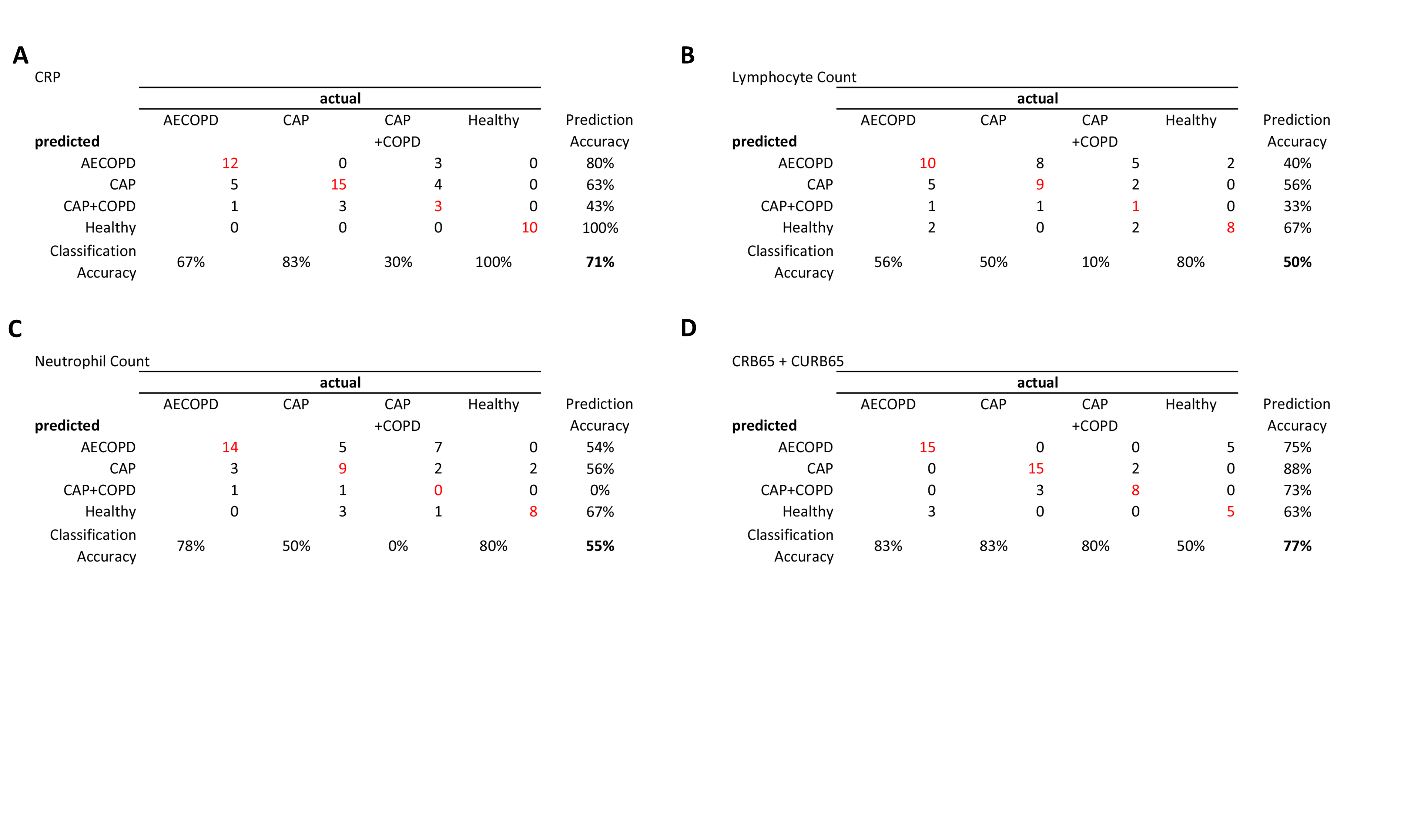

Supplement: Supplementary file 3 [file Image_2.TIF]

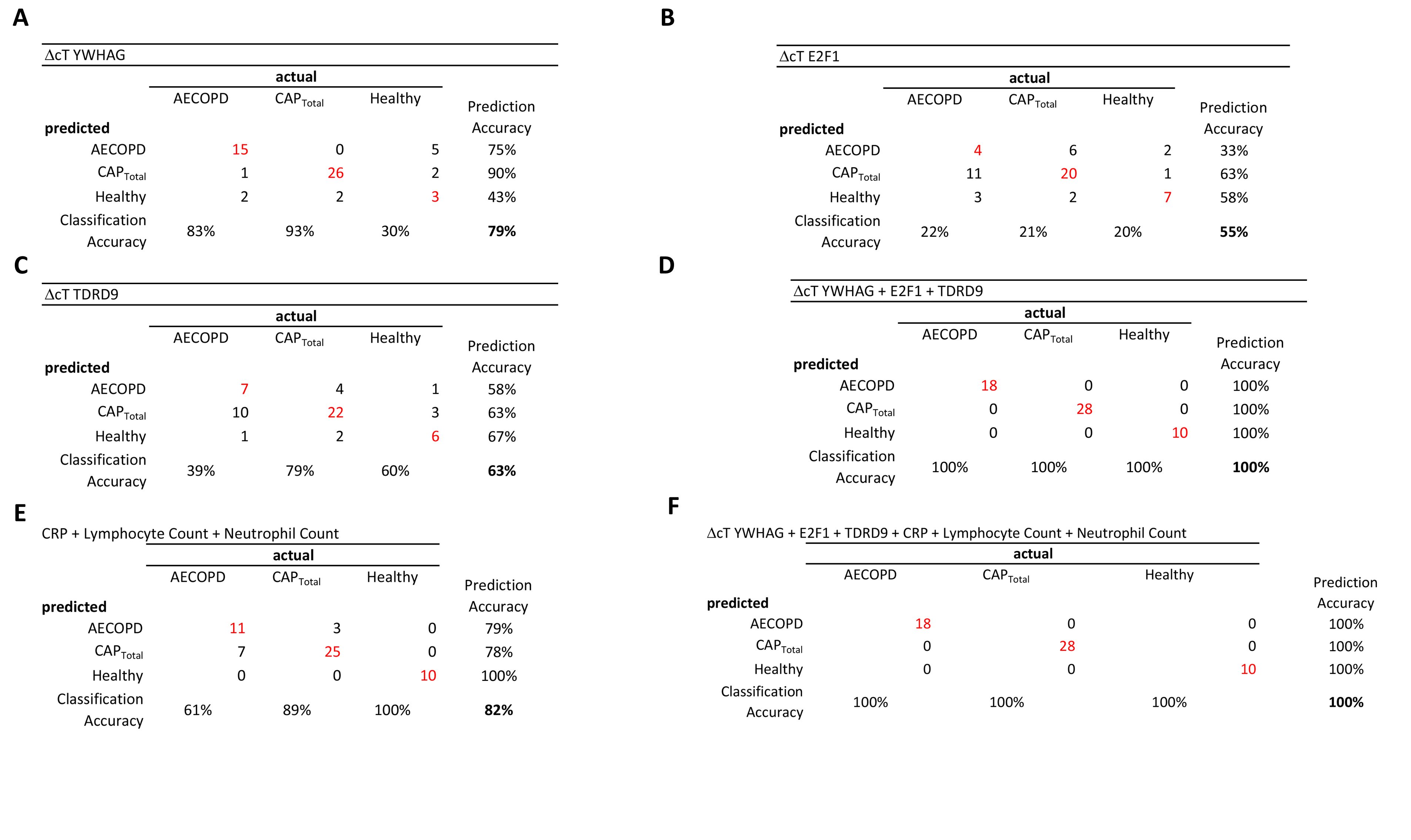

Supplement: Supplementary file 4 [file Image_3.TIF]

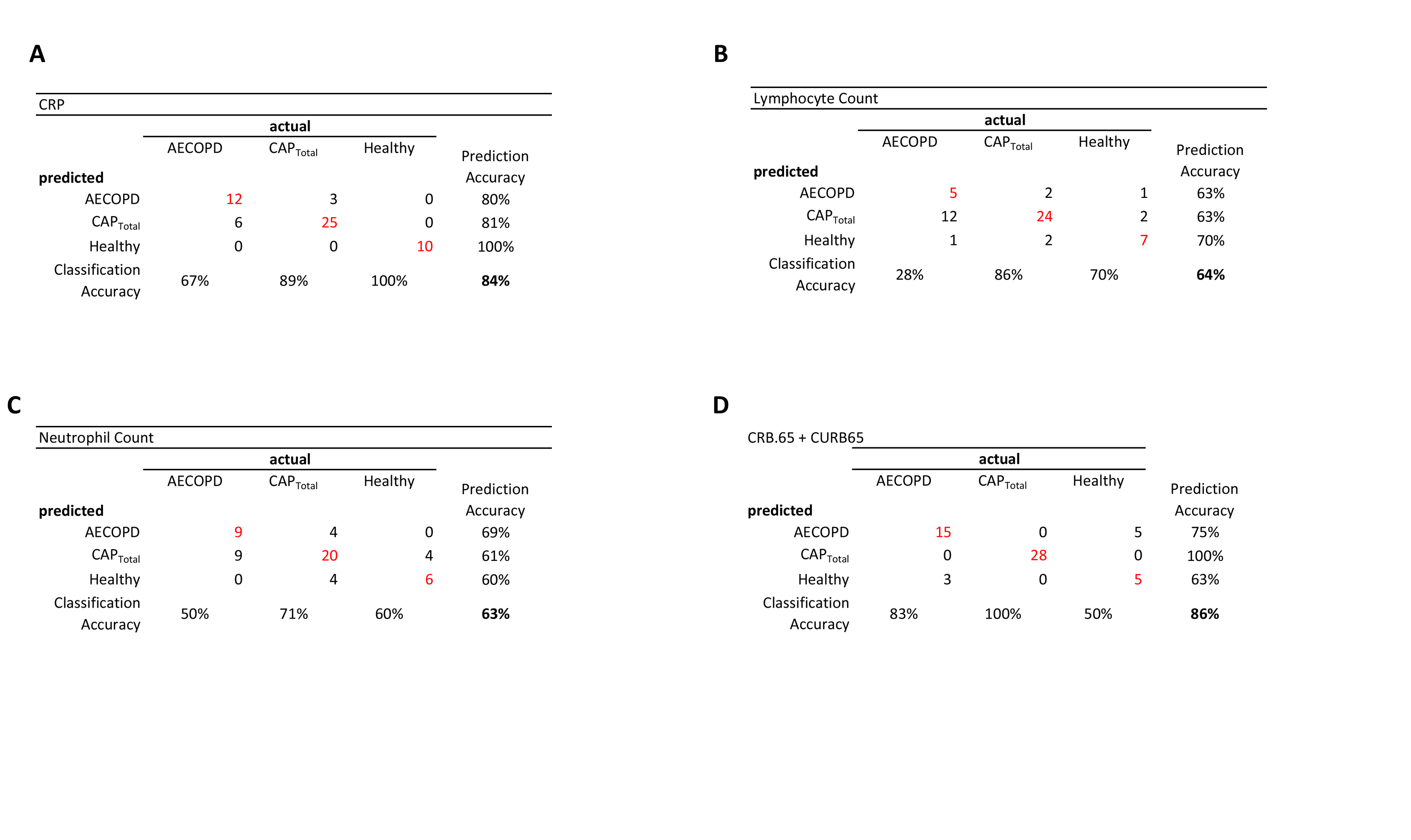

Supplement: Supplementary file 5 [file Image_4.TIF]
